# Deciphering the Genetic Association Between the FODMAP Diet and Recurring Oral Aphthae—The Mediating Role of Ferroptosis

**DOI:** 10.1002/fsn3.70949

**Published:** 2025-09-09

**Authors:** Baolin Jia, Xiaojuan Wu, Qiang Wang, Jun Ren, Guixin Li, Xianjie Zheng, Sen Yang

**Affiliations:** ^1^ Department of Oral and Maxillofacial Surgery Suining Central Hospital Suining Sichuan China; ^2^ Department of Respiratory Medicine and Critical Care Medicine Suining Central Hospital Suining Sichuan China

**Keywords:** dihydrolipoamide dehydrogenase, FODMAP diet, mendelian randomization, recurrent oral aphthae

## Abstract

The incidence of recurrent oral aphthae (ROA) is on the rise. Its etiology is unknown, particularly its relationship with the FODMAP diet. This investigation delved into their genetic correlation and elucidated the role of ferroptosis. Two‐sample Mendelian randomization (MR) analysis was performed, and mediation MR analysis to investigate the relationship between the FODMAP Diet, Ferroptosis, and ROA using GWAS data from Finngen for ROA, deCODE for ferroptosis, and UKB for the FODMAP Diet. Subsequently, a two‐step mediation analysis was conducted to examine the mediating role of Ferroptosis in the association between the FODMAP Diet and ROA. MR revealed a protective association between dried fruit and tea consumption and the risk of ROA, while poultry and beef intake showed a significant positive correlation with ROA risk. Subsequent investigation into iron‐related genes identified 10 key proteins that may mediate the impact of dietary habits on ROA development. Mediation analysis demonstrated that tea consumption indirectly influenced ROA progression by modulating Dihydrolipoamide Dehydrogenase (DLD) protein expression, accounting for 21.15% of the total effect. Sensitivity analyses confirmed our results, enhancing the statistical validity of our findingsstatistical validity. This study underscores the significance of the FODMAP Diet in preventing and treating ROA. It proposes the clinical utility of DLD as a diagnostic and therapeutic target for ROA and underscores the critical role of dietary management.

## Graphical abstract

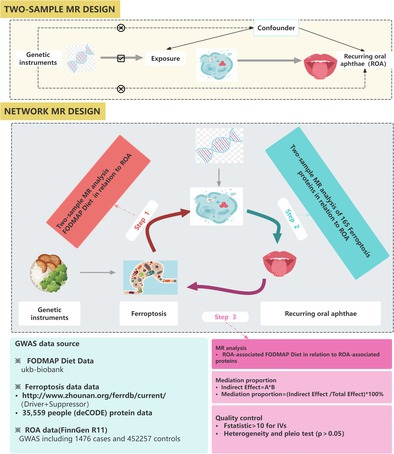

## 1 Introduction

ROA, commonly known as canker sores, is a prevalent oral mucosal disorder characterized by painful, recurring ulcers that significantly impact quality of life (Ship et al. 2000; Letsinger et al. 2005). And ROA is also considered to be a high‐risk factor for oral cavity cancer (OC) (Chen, Li, et al. 2025; Mirfendereski et al. 2025). Although the cause of the disease is not entirely clear, some studies suggest that it may be related to an inflammatory response and autoimmune function. Emerging evidence suggests a potential link between dietary triggers and ROA pathogenesis, particularly through immune modulation and gut‐oral axis interactions (Sözeri et al. 2021; Qin et al. 2018; Michailou and Perdikogianni 2025).

Fermentable oligosaccharides, disaccharides, monosaccharides, and polyols (FODMAPs)—a group of poorly absorbed short‐chain carbohydrates—have gained attention for their role in promoting low‐grade inflammation and intestinal dysbiosis (Kasti et al. 2025; Alowo et al. 2025). High‐FODMAP foods (e.g., wheat, onions, dairy) may exacerbate systemic inflammation and mucosal immune responses. In recent years, many studies have shown that the FODMAP diet is associated with a variety of gastrointestinal diseases (Cuffe et al. 2025; Zhang et al. 2025). The low‐FODMAP diet can relieve gastrointestinal diseases and improve a variety of gastrointestinal symptoms (Chu et al. 2025; Varney et al. 2025). However, no studies have been conducted to investigate the relationship between the FODMAP diet and ROA. This study aims to explore the relationship between the FODMAP diet and ROA from a genetic perspective and to explore the role of ferroptosis in this process.

## 2 Method and Materials

### 2.1 Stude Design

**FIGURE 1:**
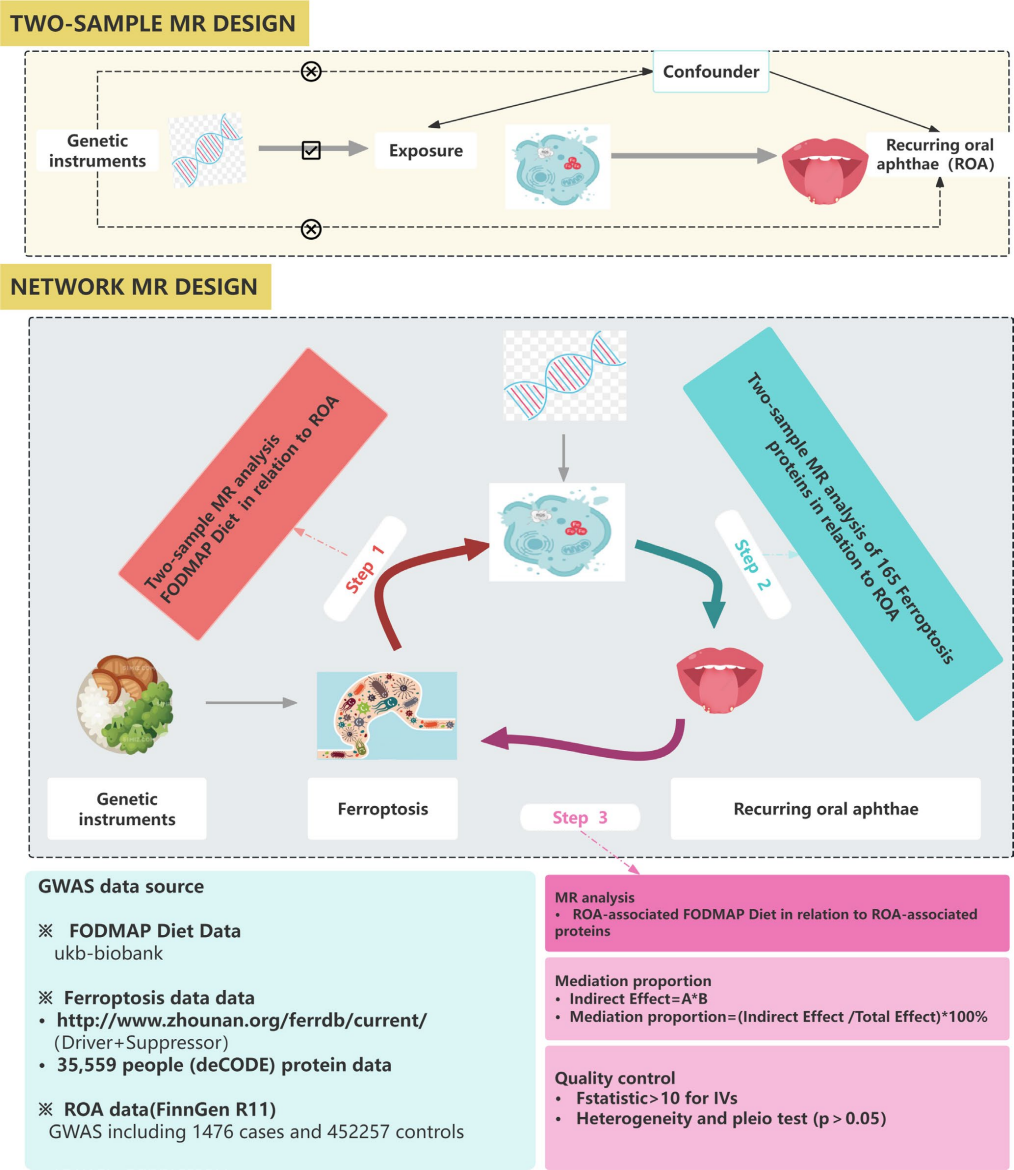
Overall design of the MR analysis framework in this study. A flow chart depicts how the MR analysis was conducted step by step in this study.

This study was reported following the Strengthening the Reporting of Observational Studies in Epidemiology Using MR guidelines (Skrivankova et al. 2021). Figure 1 illustrates the study flow. Initially, an MR analysis was conducted to explore the causal link between the FODMAP dietary framework and ROA, yielding a coefficient denoted as βc. Subsequently, the FerrDb database was used to identify Ferroptosis‐related inhibitors or drivers, and cis‐pQTLs obtained from Decode, and further MR analysis was performed to explore the association with ROA, resulting in a coefficient denoted as βb. Following this, MR investigated the correlation between the ROA‐related FODMAP diet and ROA‐related ferroptosis proteins, with the coefficient recorded as βa. Finally, the mediating role of the Ferroptosis pathway between the FODMAP diet and ROA risk was assessed using a two‐step method, where the proportional mediating effect was calculated as βa × βb/βc.

### 2.2 Data Source

Genetic associations for 28 dietary intakes were derived from a comprehensive genome‐wide association study (GWAS) dataset produced by the Medical Research Council Integrative Epidemiology Unit (MRC‐IEU) in 2018. The dietary exposures encompassed categories such as low FODMAP (e.g., bananas, broccoli, tomatoes, pork, beef, poultry, lamb, lobster/crab, fish, cheese, feta, bran cereal, tea, coffee, beer, and red wine), high FODMAP (e.g., apples, cherries, mangoes, garlic, celery, and milk), and other items (including fresh fruits, dried fruits, cooked vegetables, salads, and cereal). Detailed information on the data sources for the 28 FODMAP dietary intakes can be found in Table S1, while Table S2 provides a list of corresponding food frequency questionnaire (FFQ) queries for each dietary intake. Further specifics are available in the UK Biobank Data Showcase (http://biobank.ndph.ox.ac.uk/showcase/).

We obtained summary‐level genetic association data for 4907 circulating proteins from a large‐scale pQTL study involving 35,559 Icelandic participants. The proteomic analysis employed a multiplexed modified aptamer‐based binding assay, specifically the SOMAscan v4 technology. Detailed information on the GWAS procedures can be found in the original literature (Ferkingstad et al. 2021). Then, a total of 483 ferroptosis‐related “suppressors” and “drivers” were identified from the FerrDb dataset, of which 165 were present in the deCODE database (Table S3).

The genome‐wide association study (GWAS) data for ROA used in this study were derived from the Finngen database, a public‐private partnership project aimed at exploring founder genotype–phenotype correlations in the Finnish population (https://www.finngen.fi/en). The dataset ID is finngen_R11_K11_APHTA_RECUR.gz, comprising 1476 PD patients and 452,257 control samples. ROA diagnosis was based on International Classification of Diseases (ICD) criteria (ICD‐10 codes: K12.00–K12.03, K12.10–K12.14, K12.18), which were clinically validated through medical records and physician diagnoses. Further details can be accessed via the following link: https://r11.risteys.finregistry.fi/endpoints/K11_APHTA_RECUR.

Ethical approval and informed consent were obtained from the original study participants for all data utilized in this research.

### 2.3 Instrumental Variables Selection

We interrogated the GWAS database to identify SNPs in accordance with the three assumptions of MR. SNPs linked to each FODMAP diet were selected at a significance level of *p* < 5 × 10^−8^ to ensure their representativeness. However, this threshold did not yield SNPs for certain FODMAP diets. Subsequently, the threshold was revised to *p* < 5 × 10^−6^, accompanied by stringent clumping parameters (*r* ^2^ = 0.001 and kb = 10,000), to fulfill the minimal requirements for Mendelian randomization studies, necessitating a minimum of 10 appropriate SNPs (Gao et al. 2019). Besides, for this study, we instituted stringent inclusion criteria for pQTLs: (1) Achieved genome‐wide significance in their associations (*p* < 5 × 10^−8^); (2) Positioned externally to the major histocompatibility complex (MHC) region (chr6, 26–34 Mb); (3) Demonstrated an unequivocal independent association, adhering to an LD clumping *r* ^2^ threshold of < 0.1; KB = 10,000; (4) Classified as a cis‐acting pQTL.

### 2.4 Mediation Analysis

To investigate the association between the FODMAP diet and ROA, as well as the role of ferroptosis in mediating the link between the FODMAP diet and ROA, we performed MR and mediation analyses (Figure 1). Specifically, this approach decomposes the total effect into an indirect effect (mediated by the mediator) and a direct effect (non‐mediated). That is, the overall impact of the FODMAP diet on ROA consists of two components: (1) the direct effect of the FODMAP diet on ROA (βc); and (2) the indirect effect mediated by ferroptosis (βa × βb). By calculating the percentage of the indirect effect relative to the total effect (i.e., the ratio of the indirect effect to the total effect c), we quantified the potential mediating role of ferroptosis in the association between the FODMAP diet and ROA. Given that mediation analysis assumes the absence of unmeasured confounders, we used genetic instruments to reduce confounding and conducted sensitivity analyses to evaluate the robustness of our findings.

### 2.5 Mendelian Randomization Analysis

Causal estimates from MR analyses can only be reliably interpreted when three key assumptions are met. Multiple methods exist for causal inference in MR, and this study employed the inverse‐variance weighted (IVW) method as the primary reporting model. To validate the robustness of the findings, we conducted multiple sensitivity analyses. Specifically, Cochran's Q test was used to assess heterogeneity among genetic variants, with a *p*‐value > 0.05 indicating no significant heterogeneity. MR‐Egger regression intercept was applied to evaluate horizontal pleiotropy, where a *p*‐value > 0.05 suggested no evidence of horizontal pleiotropy. All MR analyses were performed using the R package TwoSampleMR (v 0.6.8).

## 3 Result

### 3.1 Four Types of FODMAP Diet Frame Were Potentially Associated With ROA

The *F*‐statistics for each SNP and the general *F*‐statistics were all greater than the empirical threshold of 10, suggesting that all SNPs had sufficient validity (Tables S4 and S5).

**FIGURE 2:**
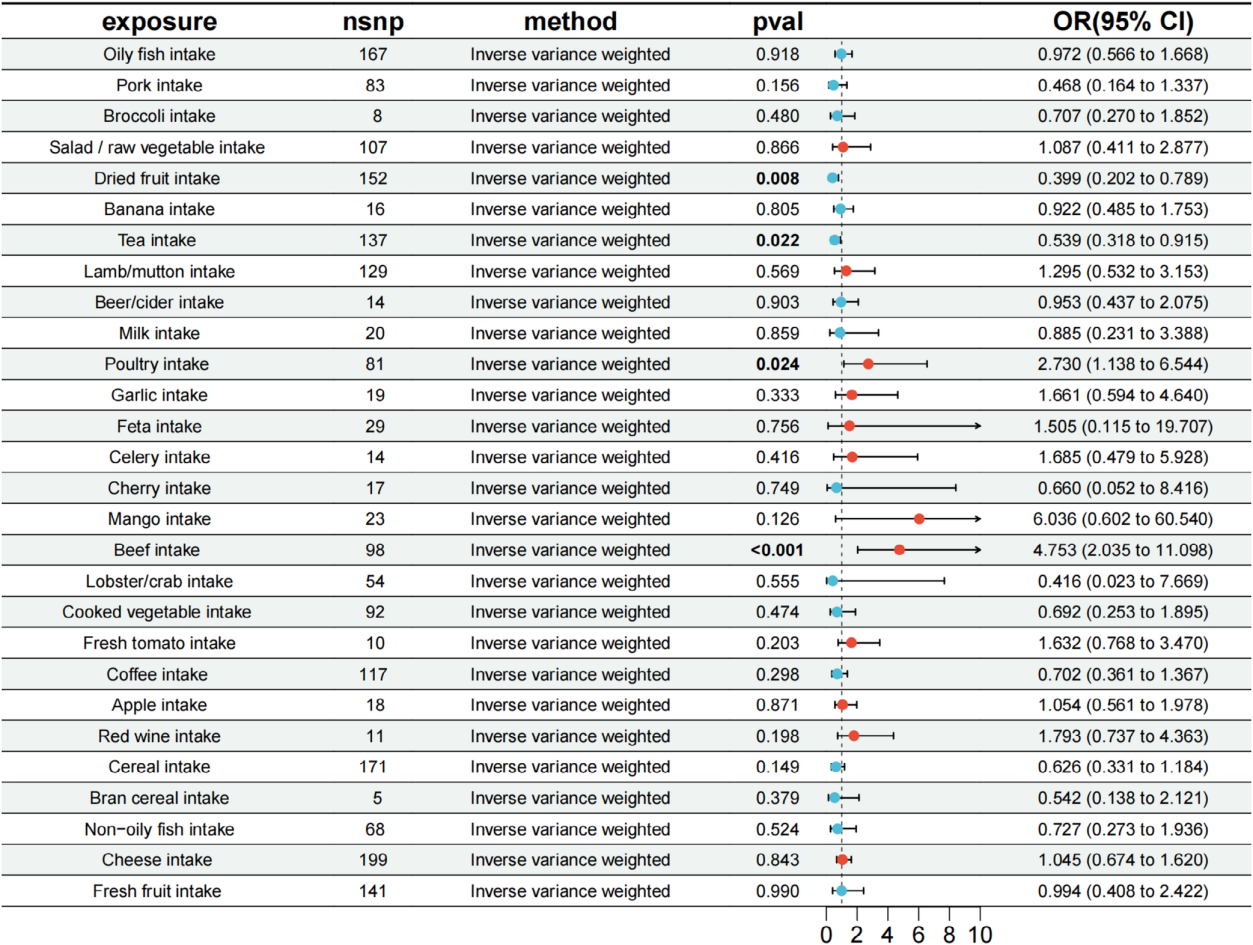
Causal estimates of FODMAP diet on ROA by MR analysis. The odds ratio (OR) was estimated using the IVW method. The horizontal bars represent 95% confidence intervals (CI).

Based on GWAS summary statistics of ROA from a Finnish database with European ancestry, we evaluated the causal association of the 28 FODMAP diet frame on ROA risk. Results showed that three Low FODMAP diets were causally associated with reduced ROA risk. Specifically, poultry intake (IVW: OR = 2.73, 95% CI: 1.138–6.544, *p* = 2.441 × 10^−2^) and beef intake (IVW: OR = 4.753, 95% CI: 2.035–11.098, *p* = 3.149 × 10^−4^) were positively correlated with ROA risk. Conversely, tea intake (IVW: OR = 0.539, 95% CI: 0.318–0.915, *p* = 2.208 × 10^−2^) was associated with decreased ROA risk (Figure 2, Table S6). Additionally, one High FODMAP diet exhibited a negative causal relationship with ROA (IVW: OR = 0.399, 95% CI: 0.202–0.789, *p* = 8.296 × 10^−3^) (Figure 2, Table S6). Heterogeneity tests revealed significant heterogeneity for cereal intake (IVW: *p*_heterogeneity_ = 0.021) (Table S6). The MR‐Egger intercept test detected no significant evidence of horizontal pleiotropy (*p*_pleiotropy_ > 0.05) (Table S6). Collectively, MR analyses support a robust conclusion that FODMAP diet components have causal associations with ROA risk.

### 3.2 Ten Ferroptosis‐Related Proteins Were Correlated With ROA

A total of 483 ferroptosis‐related molecules were obtained from the FerrDb database, of which 165 were present in the deCODE database. We retrieved the cis‐pQTLs of these 165 cis‐pQTLs and further performed MR analysis.

**FIGURE 3:**
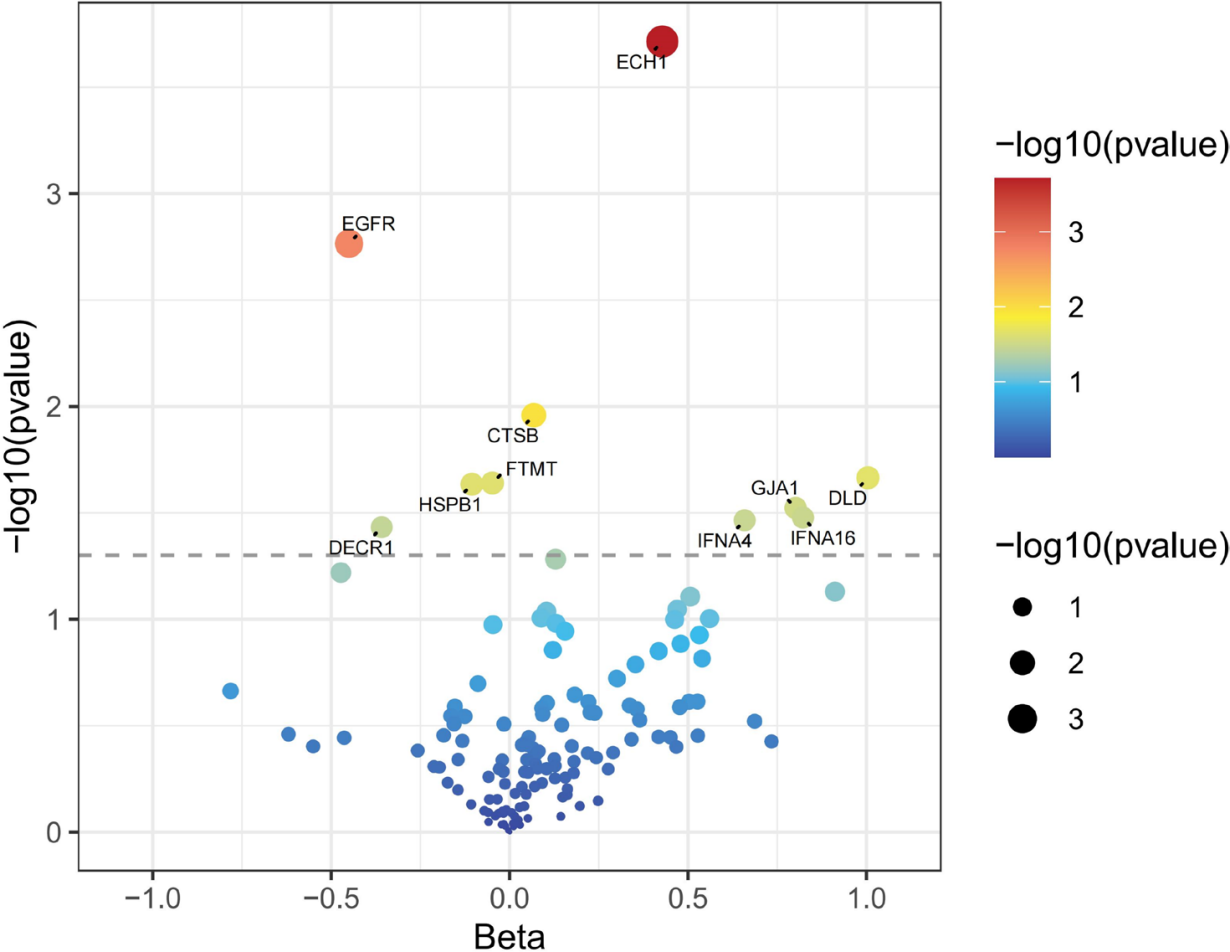
Volcano plot showing the association of ferroptosis‐related proteins with ROA.

The result showed that 10 pQTLs were causally associated with ROA risk. Specifically, IFNA4 (IVW: OR = 1.932, 95% CI: 1.05–3.554, *p* = 3.43 × 10^−2^), ECH1 (IVW: OR = 1.534, 95% CI: 1.225–1.921, *p* = 1.93 × 10^−4^), IFNA16 (IVW: OR = 2.275, 95% CI: 1.067–4.849, *p* = 3.33 × 10^−2^), GJA1 (IVW: OR = 2.228, 95% CI: 1.081–4.595, *p* = 3 × 10^−2^), CTSB (IVW: OR = 1.07, 95% CI: 1.016–1.127, *p* = 1.1 × 10^−2^), and DLD (IVW: OR = 2.73, 95% CI: 1.159–6.43, *p* = 2.16 × 10^−2^) were positively correlated with ROA risk. Conversely, HSPB1 (IVW: OR = 0.9, 95% CI: 0.821–0.986, *p* = 2.32 × 10^−2^), FTMT (IVW: OR = 0.953, 95% CI: 0.914–0.993, *p* = 2.29 × 10^−2^), EGFR (IVW: OR = 0.638, 95% CI: 0.481–0.845, *p* = 1.72 × 10^−3^), and DECR1 (IVW: OR = 0.699, 95% CI: 0.499–0.987, *p* = 3.69 × 10^−2^) was associated with decreased ROA risk (Figure 3, Table S7). Heterogeneity tests revealed significant heterogeneity for CDH1 (IVW: *p*_heterogeneity_ = 0.047) and PEBP1 (IVW: *p*_heterogeneity_ = 0.044) (Table S7).

### 3.3 Three Ferroptosis‐Related Proteins as Interventional Targets by FODMAP Diet

**FIGURE 4:**
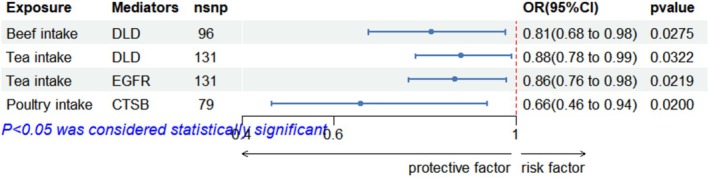
Forest plots showing the causal effects of ROA‐related FODMAP diet on FODMAP‐related ferroptosis proteins.

After identifying ROA‐related FODMAP diet frame and ROA‐related ferroptosis proteins, we further performed MR analysis to investigate the correlations between FODMAP diet and ferroptosis proteins. We found that three FODMAP diets (Beef, Tea, and Poultry intake) were associated with three ROA‐related ferroptosis molecules (DLD, EGFR, and CTSB) (Figure 4; Table S8).

### 3.4 Tea Intake Mediates the Effect of the FODMAP Diet on ROA

**FIGURE 5:**
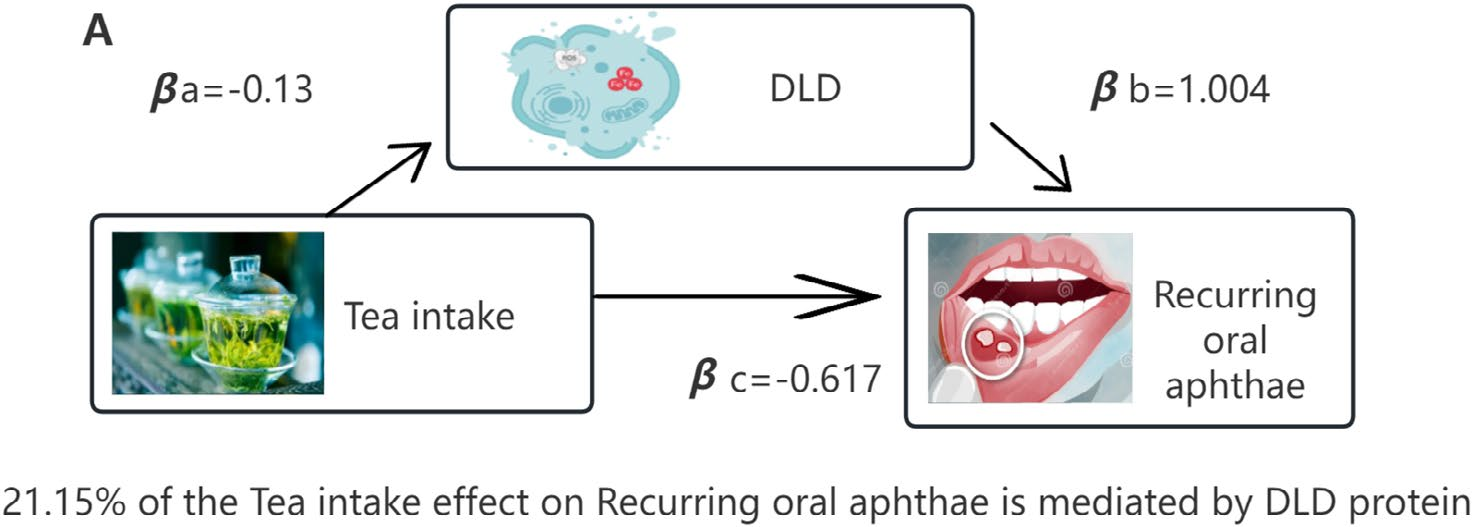
DLD mediators of the relationships between the FODMAP diet and ROA.

Figure 5 illustrates the FODMAP diet–ferroptosis–ROA pairs with mediating effects. Next, a two‐step approach was used to evaluate whether Dihydrolipoamide Dehydrogenase (DLD) acts as a mediator in the causal effect of the FODMAP diet on ROA risk (βa: the effect from Tea intake to DLD, and βb: the effect from DLD to ROA). We calculated the total effect (βc: he effect from Tea intake to ROA), mediation effect (βa × βb), and direct effect (βc' = βc − βa × βb) (Table 1). Low FODMAP Tea intake indirectly decreased ROA development by regulating DLD protein expression, with the mediating effect of Tea intake accounting for 21.15% of the total effect.

**TABLE 1:** Mediator analysis results of FODMAP diet, DLD protein expression, and ROA’s disease risk

FODMAP Diet	Ferroptosis proteins	Path a βa	Path b βb	Total effect βc	Direct effect β'c	Indirect effect βa*b	Proportion mediated (%)
Tea intake	Dihydrolipoamide dehydrogenase	−0.13	1.004	−0.617	−0.487	−0.130	21.15%

## 4 Discussion

ROA is a common inflammatory condition characterized by painful oral ulcers, affecting up to 25% of the general population. Despite its prevalence, the precise etiology remains elusive, with genetic predisposition, immune dysregulation, and dietary triggers implicated in its pathogenesis (Katebi et al. 2025; D'Amario et al. 2025). Fermentable oligosaccharides, disaccharides, monosaccharides, and polyols (FODMAPs)—a group of poorly absorbed short‐chain carbohydrates—have gained attention for their role in promoting low‐grade inflammation and intestinal dysbiosis. High‐FODMAP foods (e.g., wheat, onions, dairy) may exacerbate systemic inflammation and mucosal immune responses (Scarlata et al. 2025; Montero‐Carrasco et al. 2024). Our study provides the first evidence of a genetic association between a FODMAP (fermentable oligosaccharides, disaccharides, monosaccharides, and polyols) diet and ROA, mediated through ferroptosis—a novel iron‐dependent form of regulated cell death.

In this study, two‐sample Mendelian randomization (MR) analysis was performed and mediation MR analysis to investigate the relationship between the FODMAP Diet, Ferroptosis, and ROA using GWAS data from Finngen for ROA, deCODE for Ferroptosis, and UKB for the FODMAP Diet. MR revealed a protective association between dried fruit and tea consumption and the risk of ROA, while poultry and beef intake showed a significant positive correlation with ROA risk. Subsequent investigation into iron‐related genes identified 10 key proteins that may mediate the impact of dietary habits on ROA development. Mediation analysis demonstrated that tea consumption indirectly influenced ROA progression by modulating DLD protein expression, accounting for 21.15% of the total effect. Sensitivity analyses confirmed our results, enhancing our findings' statistical validity.

DLD is an important gene in ferroptosis. Many studies have confirmed its role in a variety of diseases (Hammann et al. 2025; Mailloux 2025; Hu et al. 2025). Ferroptosis is characterized by iron accumulation, glutathione depletion, and uncontrolled lipid peroxidation, ultimately leading to plasma membrane rupture and inflammatory cell death (Liu et al. 2025; Lei et al. 2025). High‐FODMAP diets, particularly those rich in fructans and polyols, have been linked to intestinal inflammation and oxidative stress in irritable bowel syndrome (IBS) patients. The high FODMAP diet may cause ferroptosis of cells in vivo by affecting oxidative stress and iron metabolism (Narimani et al. 2024; Muscogiuri et al. 2024). Recent studies have found that intestinal dysbiosis is associated with ferroptosis, and the FODMAP diet can significantly change the composition of intestinal flora and thus affect ferroptosis. Keratinocyte death due to ferroptosis can disrupt oral mucosal integrity (Chen et al. 2017). The products of lipid peroxidation that accompany ferroptosis can also directly damage epithelial cells. Moreover, iron overload can promote the generation of reactive oxygen species (ROS) and aggravate the local inflammatory response (Yang et al. 2025; Oh et al. 2024). These reasons may be the theoretical basis for the mediating role of ferroptosis between the FODMAP diet and ROA.

This study found that dried fruit and tea drinking had negative regulatory effects on the occurrence of ROA. This may imply that restricting dietary intake of FODMAP may reduce oxidative stress and inflammation, suggesting its potential benefits in ROA. Ferroptosis was found to be a key mediator between the FODMAP diet and ROA, suggesting that ferroptosis inhibitors may have a potential role in the prevention or treatment of ROA. At the same time, the genetic analysis of this study helps to screen patients with ROA who can benefit from dietary modification or targeted ferroptosis therapy. Drinking tea is a good dietary habit for people prone to ROA. Epigallocatechin gallate (EGCG) is a component found in tea. At present, the onset of RAS is believed to be related to oxidative damage of the oral mucosa, excessive inflammatory responses mediated by immune cells, and the effects of oral microorganisms, etc. Among the components of tea, EGCG may be the effective ingredient in reducing the risk of RAS, because EGCG has been found to have the ability to eliminate free radicals and alleviate oxidative damage to the mucous membranes. EGCG is believed to be able to reduce the production of pro‐inflammatory factors, and it can also directly inhibit some harmful microorganisms. All these effects are closely related to the reduction of RAS risk (Zhang et al. 2025; Chen, Wang, et al. 2025; Wang et al. 2024). This is also consistent with the results of our current study.

Our study has several limitations. First, the GWAS data mainly included participants from European populations, which may have introduced racial bias. Secondly, the onset and progression of ROA are affected by many factors. Therefore, the results of the present study can only partially elucidate the relationship between the FODMAP diet and ROA. In addition, the conclusions of this study need to be further verified by basic research. In future research, we hope to conduct more clinical studies to verify the credibility of the conclusions of this research. By implementing a prospective intervention on the FODMAP diet for individuals at high risk of ROA, we can determine whether the FODMAP diet is associated with the risk of ROA. It is expected that this approach can be clinically applied to reduce the risk of ROA occurrence, thereby achieving the goal of disease prevention. Furthermore, we will also conduct basic experiments to explore the role of DLD as a therapeutic target for ROA.

## 5 Conclusion

In conclusion, this study underscores the significance of the FODMAP Diet in preventing and treating ROA. It proposes the clinical utility of DLD as a diagnostic and therapeutic target for ROA and underscores the critical role of dietary management.

## Author Contributions

**Baolin Jia:** conceptualization (equal), data curation (equal), formal analysis (equal), investigation (equal), methodology (equal), resources (equal), software (equal), validation (equal), visualization (equal), writing – original draft (lead). **Xiaojuan Wu:** conceptualization (equal), data curation (equal), formal analysis (equal), resources (equal), software (equal). **Qiang Wang:** conceptualization (equal), data curation (equal), formal analysis (equal), visualization (equal). **Jun Ren:** conceptualization (equal), data curation (equal), formal analysis (equal), validation (equal). **Guixin Li:** data curation (equal), formal analysis (equal). **Xianjie Zheng:** data curation (equal), formal analysis (equal). **Sen Yang:** funding acquisition (equal), project administration (equal), supervision (equal), writing – review and editing (equal).

## Ethics Statement

The authors have nothing to report.

## Consent

The authors have nothing to report.

## Conflicts of Interest

The authors declare no conflicts of interest.

## Supporting information


**Tables S1‐S8:** fsn370949‐sup‐0001‐Tables.xlsx.

## Data Availability

The original contributions presented in the study are included in the article/Supporting Information. All data are publicly available.
